# Supplementary material for: Emergent constraints on future projections of the western North Pacific Subtropical High
Source: Nat Commun. 2020 Jun 4;11:2802. doi: 10.1038/s41467-020-16631-9 (PMC7272422; doi:10.1038/s41467-020-16631-9)
Supplement: Supplementary file 1 — Supplementary Information [file 41467_2020_16631_MOESM1_ESM.pdf]

Supplementary Information

Emergent constraints on future projections of the western North Pacific Subtropical High

Chen et al.

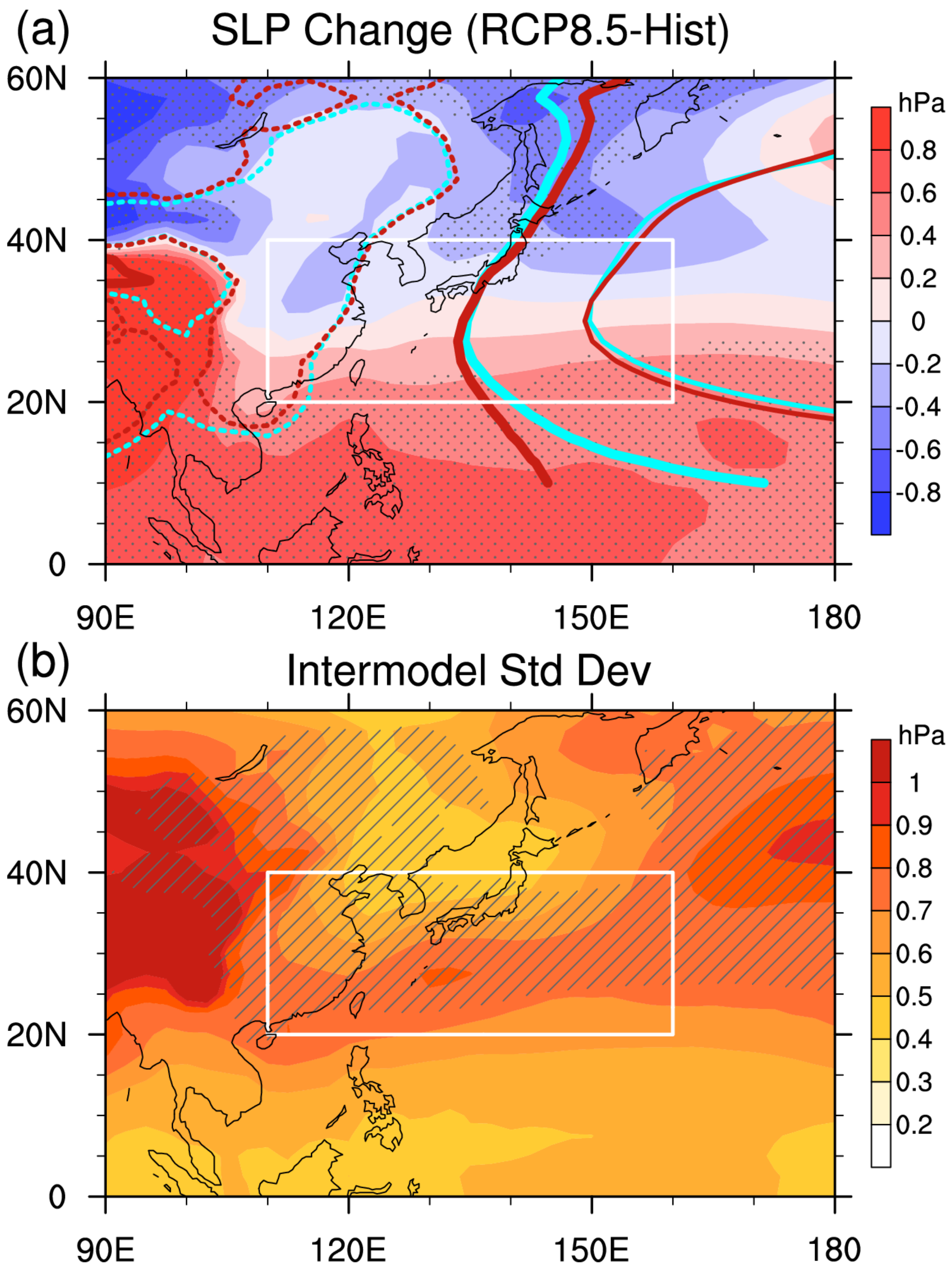

Supplementary Fig. 1: Uncertainty in projection of the western Pacific Subtropical High. a shows projected sea level pressure (SLP; hPa) changes over the East Asia and western North Pacific under the RCP8.5 scenario (2050–2099) relative to historical simulation (1956–2005). Contours are climatological SLP in the historical simulation (cyan lines) and future projection (dark red lines), drawn for 1006 (dashed lines), 1010 (thick solid lines) and 1014 (thin solid lines) hPa. Dotted regions denote more than 70% models with the same sign. **b** shows intermodel standard deviation (σ; hPa) of projected SLP changes. Hatched regions denote signal-to-noise ratio ($\bar{X}/\sigma_X$) less than 0.5. Prominent intermodel uncertainty of projected changes in SLP along the western Pacific Subtropical High ridge (white box) is manifested.

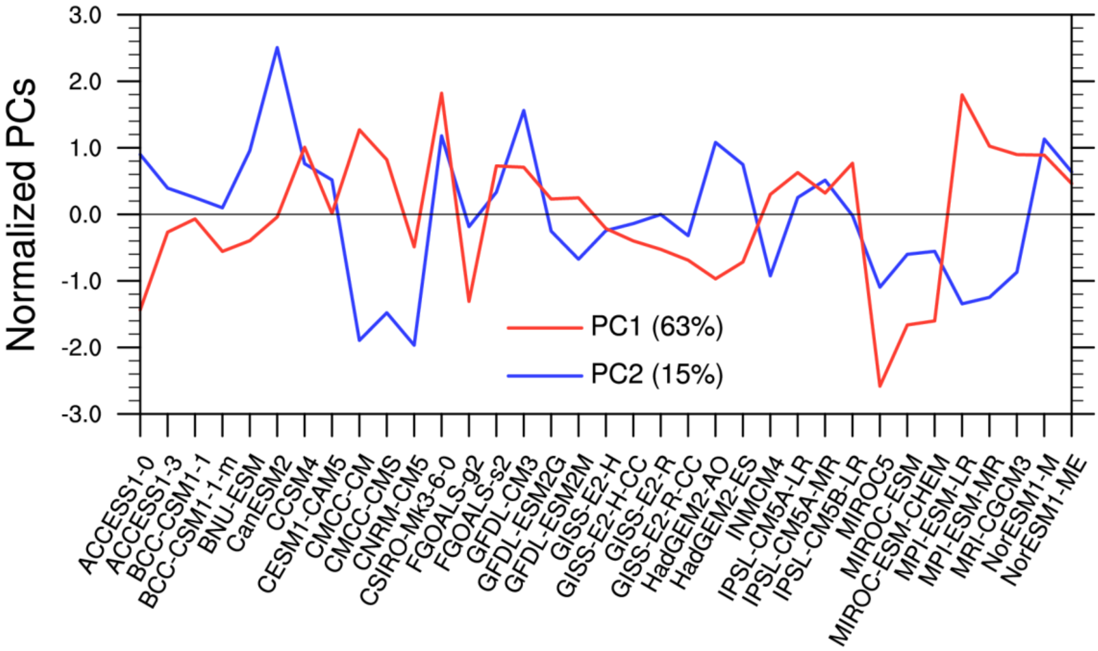

Supplementary Fig. 2: Normalized Principal Components. Here shows the first and second Principal Components (PC1 and PC2), representing two leading modes of intermodel uncertainty in projected changes of the western Pacific Subtropical High. X-axis is for 35 CMIP5 models (see Supplementary Table 1). Percentages in parentheses denote the explained intermodel variance for corresponding mode.

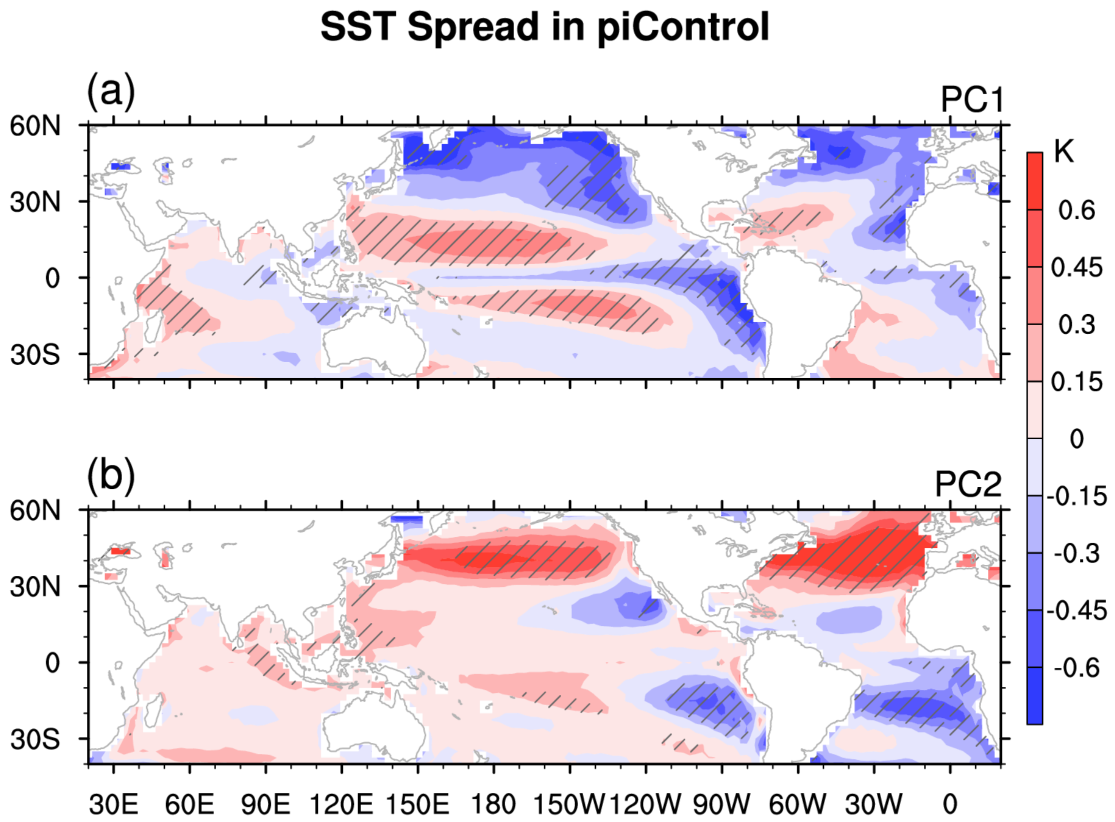

Supplementary Fig. 3: Spread pattern of sea surface temperature in pre-industrial run. a shows intermodel regression of sea surface temperature (SST; K) onto the first Principal Component (PC1) across 35 models and **b** for regression onto the second Principal Component (PC2). Hatched regions are statistically significant at the 5% level under student *t*-test. In pre-industrial run, SST spread across models is independent of uncertainty in response to external forcing.

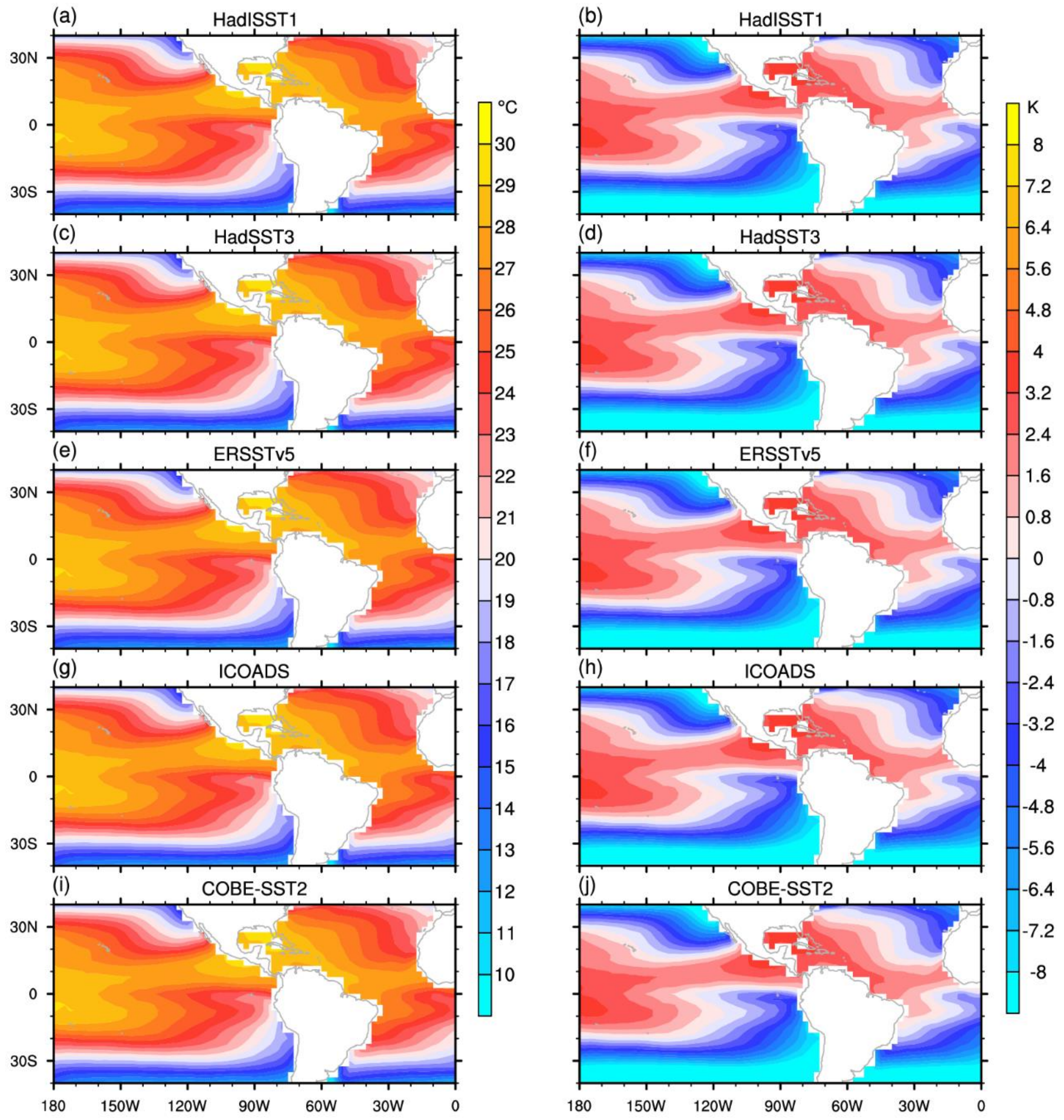

Supplementary Fig. 4: Climatological sea surface temperature in observational datasets. a, **c**, **e**, **g** and **i** are for absolute sea surface temperature (SST; °C) during 1956-2005 and **b**, **d**, **f**, **h**, and **j** for relative SST (K) by removing the mean SST in 30°S-30°N globally. All the five observational datasets are interpolated onto the 2.5°×2.5° grid. Pattern correlation coefficient between any of these data for both absolute and anomalous fields is over 0.999, showing high consistence across the observational SST datasets.

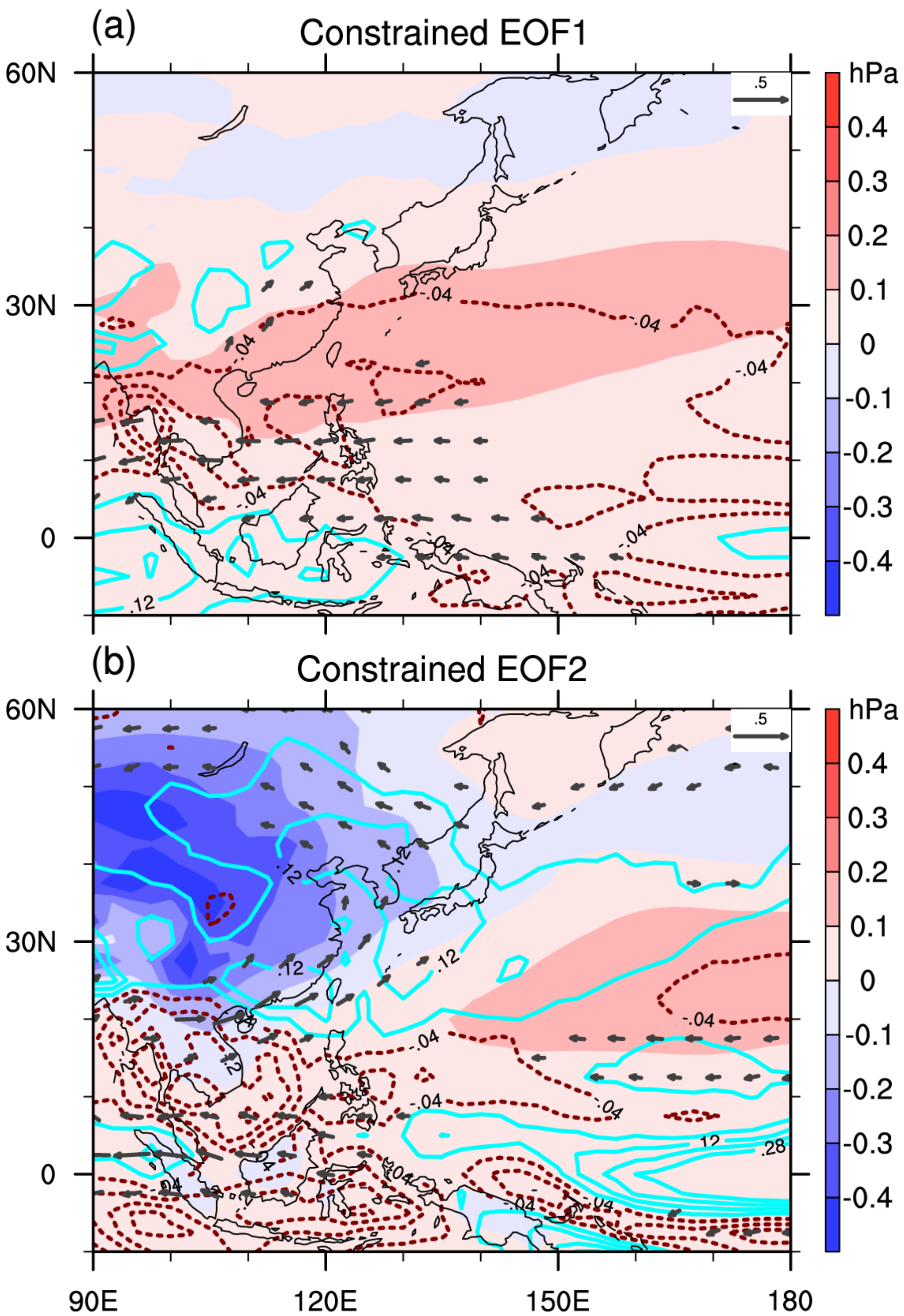

Supplementary Fig. 5: Constrained leading uncertainty modes and related circulation and precipitation. a and **b** are the two constrained leading Empirical Orthogonal Function (EOF) modes (EOF1 and EOF2) of intermodel sea level pressure changes (shadings; hPa), based on the optimal first and second Principal Components (PC1 and PC2; Fig. 2), respectively, as well as associated changes in precipitation (contours; mm day^{-1}) and wind at 850 hPa (vectors drawn for larger than 0.1 m s^{-1}). Sum of the constrained EOF1 and EOF2 is used to correct projection biases in the original results of multi-model ensemble mean (see Fig. 4b).

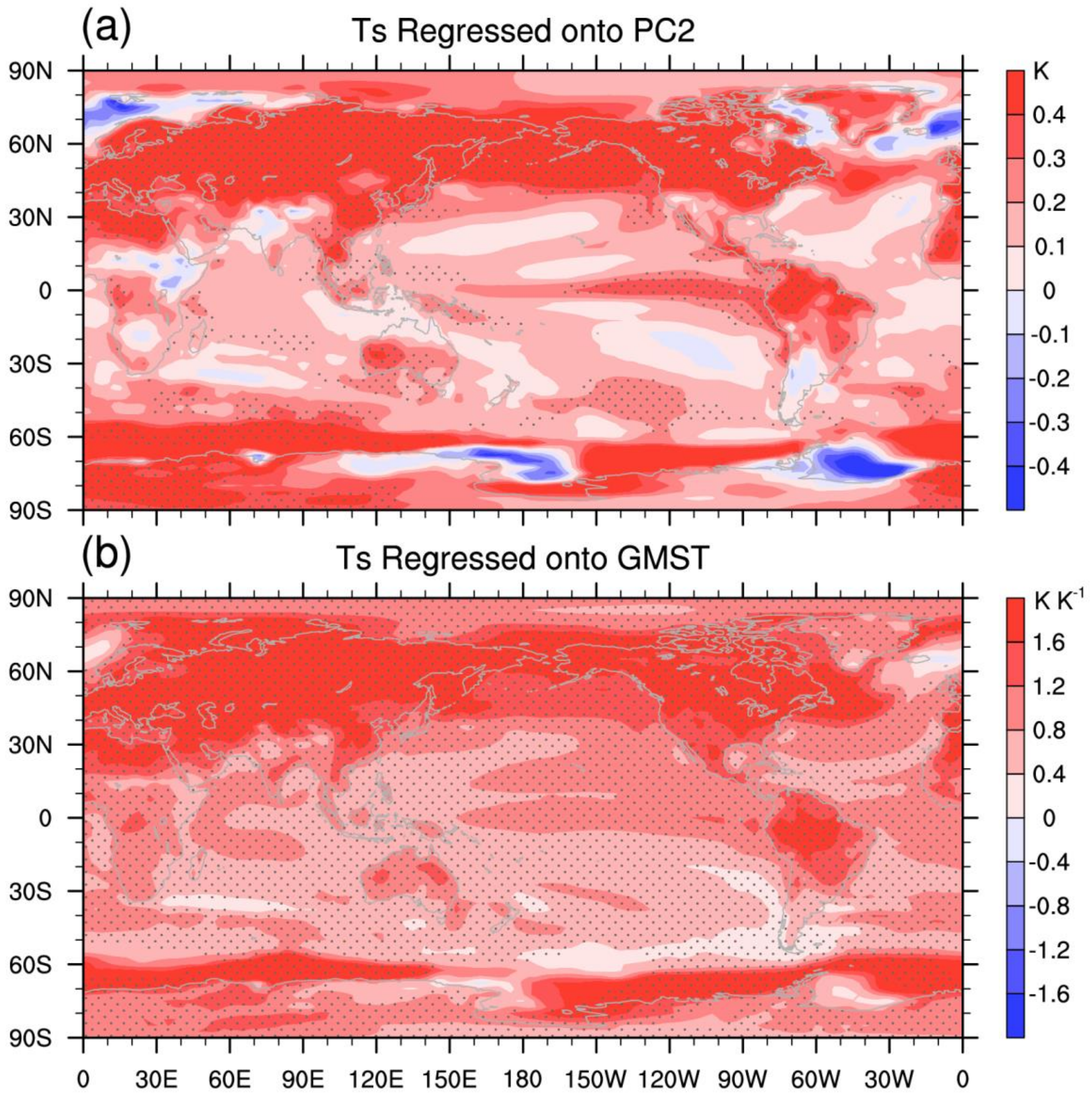

Supplementary Fig. 6: Spread patterns of projected surface temperature changes. a shows intermodel regression of projected surface temperature (Ts; K) changes under RCP8.5 in boreal summer onto the second Principal Component (PC2) across 35 models and **b** for regression (K K^{-1}) onto global mean surface air temperature (GMST) changes. Pattern correlation coefficient is 0.88. Dotted regions are statistically significant at the 5% level under student *t*-test.

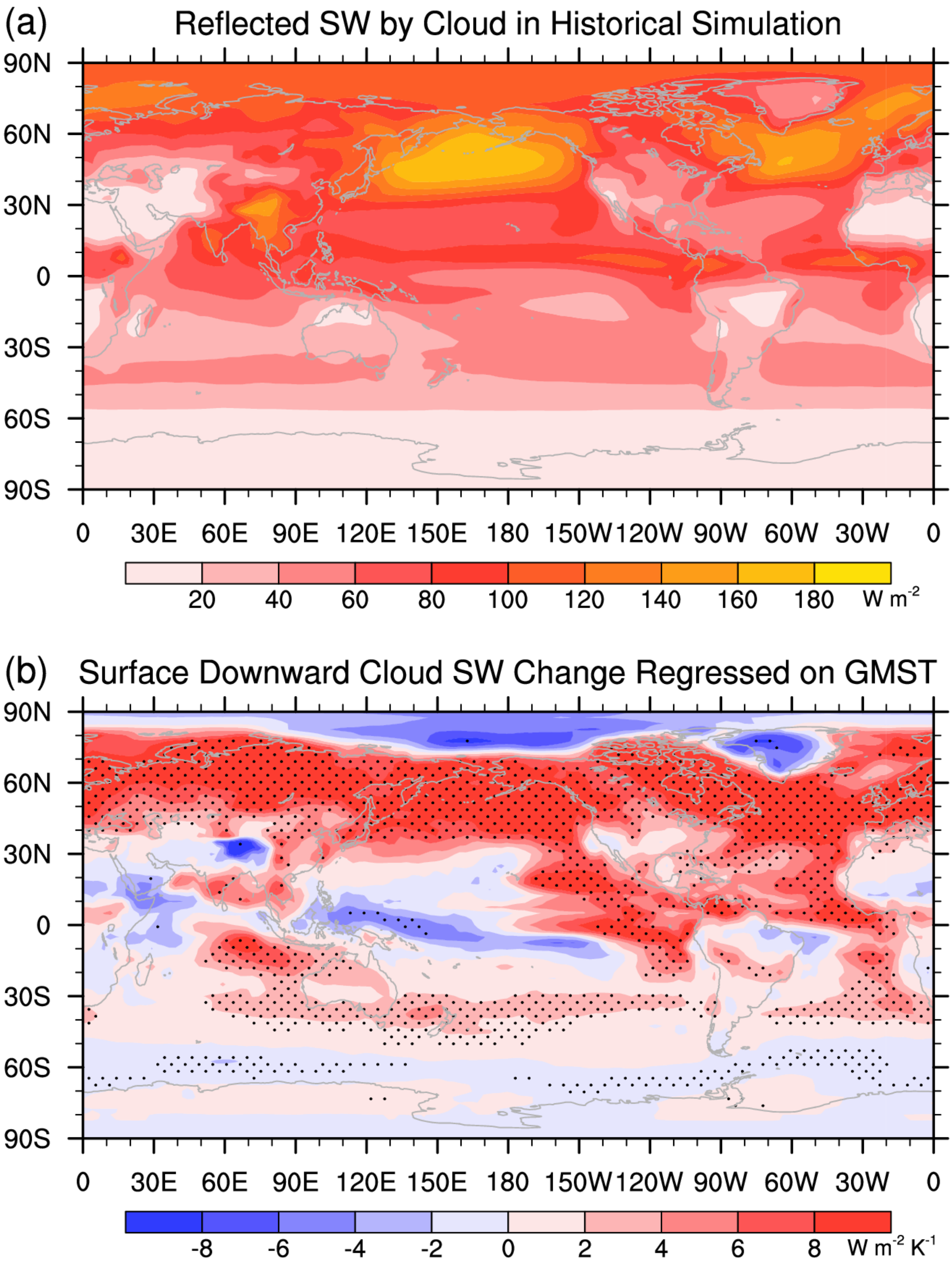

Supplementary Fig. 7: Shortwave cloud radiation mean state and spread in future changes. a shows geographical distribution of shortwave (SW; W m^{-2}) reflected by cloud in historical simulation of the multi-model ensemble mean (1956-2005) in boreal summer. **b** shows surface downward cloud shortwave change regressed on intermodel global mean surface air temperature (GMST) change (W m^{-2} K^{-1}) under RCP8.5 scenario. Dotted regions are statistically significant at the 5% level under student *t*-test. Large spread in shortwave cloud radiation changes associated with global mean warming appears in the regions where there are considerable shortwave reflective clouds, including the marine stratocumulus regions.

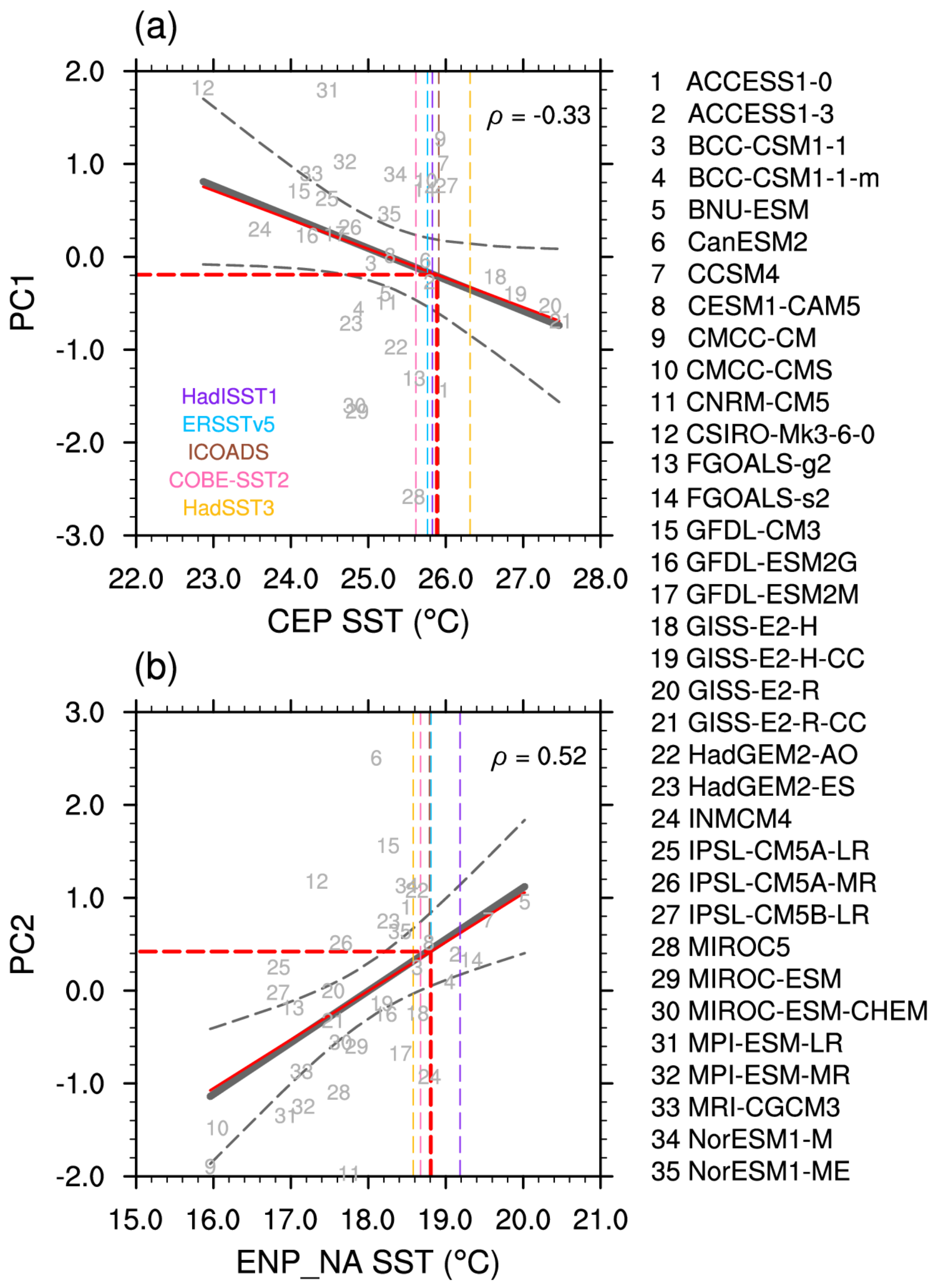

Supplementary Fig. 8: Relationship between spread in projection and historical temperature. Equatorial central-eastern Pacific (CEP) sea surface temperature (SST; °C) in **a** is an index defined in 2°S–2°N, 180–80°W (Methods) and the eastern North Pacific and North Atlantic (ENP_NA) SST index (°C) in **b** is averaged in 35–45°N, 180–10°W. Correlation coefficients (ρ) are statistically significant at the 5% level under student *t*-test. Bold grey fitting line is obtained by the least square method while thin red line is an observational correction based on Equation (5) in Methods. Black dashed curves denote the 95% confidence range of the linear regression. The indices from five observational SST datasets (HadISSTv1.1, ERSSTv5, ICOADS, COBE-SST2 and HadSST3; vertical thin dashed lines) are used to constrain the values of PCs. Mean of the five observational results yields the optimal constraints (red dashed line): PC1 = –0.19±0.95 and PC2 = 0.42±0.86.

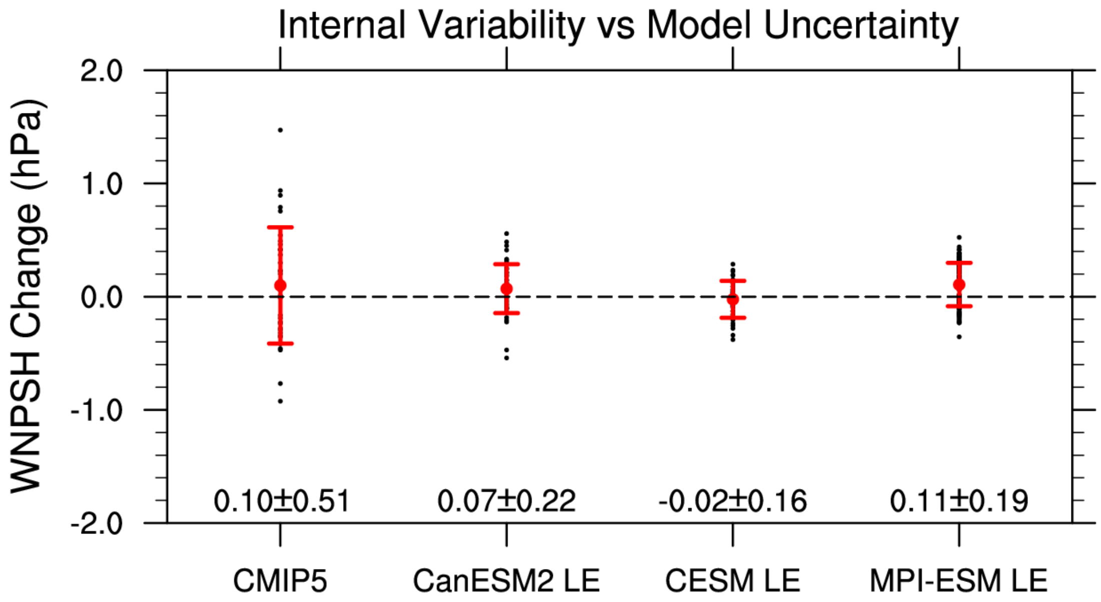

Supplementary Fig. 9: Different uncertainty sources of the western Pacific Subtropical High projection. Projected change of the western Pacific Subtropical High (WNPSH; hPa) is defined by sea level pressure difference between 2050-2099 and 1956-2005 averaged in 20–40°N, 110–160°E in 35 CMIP5 models and in large ensemble (LE) simulations of three single models, that is CanESM2 (50 members), CESM (40 members) and MPI-ESM (100 members), under the RCP8.5 scenario. Spread across models from the fifth Phase of Coupled Model Intercomparison Project (CMIP5) represents uncertainty due to different model structures. The LE experiment configuration for members in the same model only differs from initial conditions. Spread ($\pm 1\sigma$) of the WNPSH changes in a LE, caused by internal variability, is less than half of the spread caused by model structure. If measured by variance ratio ($\sigma^2_{\mathrm{internal}}/\sigma^2_{\mathrm{intermodel}}$), the contributions from internal variability is less than 20%. Data sources: Historical simulations of MPI-ESM ensemble were performed with the Swiss National Computing Centre (CSCS), and the corresponding RCP8.5 simulations were performed with the facilities at the German Climate Computing Centre (DKRZ). The MPI-ESM large ensemble data are available for noncommercial use via https://esgf-data.dkrz.de/projects/mpi-ge/. Detailed information of the data and references can be found at www.mpimet.mpg.de/en/grand-ensemble/. The CanESM2 ensemble data are shared by the Canadian Center for Climate Modeling and Analysis (https://open.canada.ca/data/en/dataset/aa7b6823-fd1e-49ff-a6fb-68076a4a477c), and the CESM large ensemble data by the National Center for Atmospheric Research (http://www.cesm.ucar.edu/projects/community-projects/LENS/).

Supplementary Table 1. Basic information of 35 models from the fifth Phase of Coupled Model Intercomparison Project (CMIP5) used in this study

Model Name	Institute/Country	Atmos. Resolution (lat × lon, level)	Ocean Resolution (lat × lon, level)
ACCESS1-0	CSIRO-BOM/Australia	145 × 192, L38	300 × 360, L50
ACCESS1-3	CSIRO-BOM/Australia	145 × 192, L38	300 × 360, L50
BCC-CSM1-1	BCC/China	64 × 128, L26	232 × 360, L40
BCC-CSM1-1-m	BCC/China	160 × 320, L26	232 × 360, L40
BNU-ESM	BNU/China	64 × 128, L26	200 × 360, L50
CanESM2	CCCma/Canada	64 × 128, L35	192 × 256, L40
CCSM4	NCAR/USA	192 × 288, L27	384 × 320, L60
CESM1-CAM5	NSF-DOE-NCAR/USA	192 × 288, L27	384 × 320, L60
CMCC-CM	CMCC/Italy	240 × 480, L27	149 × 182, L31
CMCC-CMS	CMCC/Italy	96 × 192, L95	149 × 182, L31
CNRM-CM5	CNRM-CERFACS/France	128 × 256, L31	292 × 362, L42
CSIRO-Mk3-6-0	CSIRO-QCCCE/Australia	96 × 192, L18	189 × 192, L31
FGOALS-g2	IAP-THU/China	60 × 128, L26	196 × 360, L30
FGOALS-s2	IAP-LASG/China	64 × 128, L26	196 × 360, L30
GFDL-CM3	NOAA-GFDL/USA	90 × 144, L48	200 × 360, L50
GFDL-ESM2G	NOAA-GFDL/USA	90 × 144, L24	210 × 360, L63

GFDL-ESM2M	NOAA-GFDL/USA	90 × 144, L24	200 × 360, L50
GISS-E2-H	NASA-GISS/USA	89 × 144, L40	180 × 360, L26
GISS-E2-H-CC	NASA-GISS/USA	89 × 144, L40	180 × 360, L26
GISS-E2-R	NASA-GISS/USA	89 × 144, L40	180 × 288, L32
GISS-E2-R-CC	NASA-GISS/USA	89 × 144, L40	180 × 288, L32
HadGEM2-AO	KMA-NIMR/South Korea	144 × 192, L38	216 × 360, L40
HadGEM2-ES	MOHC/UK	144 × 192, L38	216 × 360, L40
INMCM4	INM/Russia	120 × 180, L21	340 × 360, L40
IPSL-CM5A-LR	IPSL/France	96 × 96, L39	149 × 182, L31
IPSL-CM5B-LR	IPSL/France	96 × 96, L39	149 × 182, L31
IPSL-CM5B-LR	IPSL/France	96 × 96, L39	149 × 182, L31
MIROC5	MIROC/Japan	128 × 256, L40	224 × 256, L50
MIROC-ESM	MIROC/Japan	64 × 128, L80	192 × 256, L44
MIROC-ESM-CHEM	MIROC/Japan	64 × 128, L80	192 × 256, L44
MPI-ESM-LR	MPI-M/Germany	96 × 192, L47	220 × 256, L40
MPI-ESM-MR	MPI-M/Germany	96 × 192, L96	404 × 802, L40
MRI-CGCM3	MRI/Japan	160 × 320, L48	368 × 360, L51
NorESM1-M	NCC/Norway	96 × 144, L26	384 × 320, L53
NorESM1-ME	NCC/Norway	96 × 144, L26	384 × 320, L53
